# Editorial: Invertebrate brains as model systems for learning, memory, and recall: development, anatomy and function of memory systems

**DOI:** 10.3389/fphys.2024.1545795

**Published:** 2025-01-07

**Authors:** André Fiala, Hagar Meltzer, Michael Schleyer, Oriane Turrel, Annekathrin Widmann

**Affiliations:** ^1^ Department of Molecular Neurobiology of Behavior, University of Göttingen, Göttingen, Germany; ^2^ Department of Molecular Cell Biology, Weizmann Institute of Science, Rehovot, Israel; ^3^ Department of Molecular Neuroscience, Weizmann Institute of Science, Rehovot, Israel; ^4^ Institute for the Advancement of Higher Education, Hokkaido University, Sapporo, Japan; ^5^ Department of Genetics, Freie Universität Berlin, Berlin, Germany

**Keywords:** learning and memory (neurosciences), invertebrate brains, computational modelling, mushroom body, navigation, autophagy, neonicotinoid insecticides, olfactory coding

One of the most fundamental scientific challenges of our time is understanding how memory systems integrate present sensory stimuli, past experiences, and future behavioral options. Modern neuroscience has made remarkable progress by examining the properties of single neurons, their synapses, and the circuits they form. Yet, a deeper understanding of learning, memory formation, and recall requires an integrative approach—one that spans molecular processes, neural circuits, and whole-organism behavior. For example, how insects integrate the smell of a food source with past experiences of successful feeding sites identified through learned odor cues—mediated by the mushroom body, a key neural circuit involved in decision-making and memory retrieval—illustrates the complexity of these processes.

In this context, invertebrate model systems offer unique advantages. Combining neural simplicity with rich behavioral repertoires and exceptional experimental accessibility, they serve as ideal platforms for uncovering fundamental principles of learning and memory. Despite having relatively small brains, invertebrates exhibit complex behaviors driven by well-defined neural circuits, making them invaluable for elucidating mechanisms of adaptive behavior. The contributions of invertebrate neuroscience extend beyond species-specific insights. Findings from these systems often reveal universal brain mechanisms, offering paradigms applicable across the animal kingdom, including vertebrates and humans, such as conserved molecular pathways and similar neuronal circuit motifs. Therefore, the impact of invertebrate neuroscience extends beyond basic science. Insights from these models can potentially inspire advancements in fields such as robotics and machine-based learning algorithms. Thus, invertebrate model systems remain a cornerstone of neuroscience research, offering unparalleled opportunities to decipher the complexities of brain function and behavior.

This Research Topic presents a collection of articles that explore how learning and memory can be studied across biological scales using invertebrate models. It features original research from diverse disciplines, including neuroanatomy, neuroethology, learning psychology, computational modeling, and neurophysiology. Together, these contributions highlight the power of studies using invertebrates in uncovering the fundamental principles of memory and learning in biological systems.

Strube-Bloss et al. used the honeybee *Apis mellifera* to investigate how visual and olfactory cues interact during associative learning. By measuring muscle activity linked to the proboscis extension response, they examined how bees respond to learned odors, lights, and combined stimuli. Their findings suggest that sensory interactions involve both acceleration and deceleration of responses, depending on the sensory modality involved. This study highlights how invertebrate models can be used to explore fundamental concepts of classical learning psychology.

A review article by Wu et al. highlights how invertebrate brains provide valuable insights into the cellular mechanisms of learning and memory, with a focus on autophagy—a process that degrades intracellular components to manage stress and maintain cellular health. While often studied in mammals, autophagy is an evolutionarily conserved process that can be conveniently explored in invertebrates. The review discusses how post-translational modifications regulate autophagy in insects such as *Bombyx mori* and *Drosophila melanogaster*. A deeper understanding of these mechanisms could enable autophagy-based intervention strategies and uncover conserved roles in cognitive processes across species.

Matsumoto et al. investigated the physiological and molecular basis of learning using the well-studied cricket *Gryllus bimaculatus*, known for its strong learning abilities. Through behavioral experiments and pharmacological interventions, they demonstrated that nicotinic acetylcholine receptors and the NO-cGMP signaling pathway play crucial roles in long-term memory formation. Their findings provide valuable insights into the molecular mechanisms underlying memory processes.

Along similar lines, Schulz et al. examined the role of nicotinic acetylcholine receptors in learning and memory as well, but in a different context—specifically, the impact of neonicotinoid insecticides. As the largest class of insecticides, neonicotinoids block these receptors, and the authors investigated how sublethal doses of these chemicals affect the learning and memory performance of both larval and adult *Drosophila melanogaster*. Additionally, they leveraged the genetic tools available for this model organism to explore the effects of neonicotinoids on synaptic integrity and transmitter release. This study demonstrates how basic research on learning and memory can inform applied research in fields like pesticide toxicity.

The mushroom body, a higher-order central brain circuit in insects, has long been linked to learning and memory but has recently been proposed to support visual navigation as well. Ants, known for their remarkable navigational skills, exemplify this possibility. Jesusanmi et al. used computational modeling to demonstrate that the mushroom body’s well-mapped connectivity could indeed support visual navigation and the learning of relevant visual cues. As the authors aptly state, “Understanding the neural basis of this behavior will provide insight into how neural circuits are tuned to rapidly learn behaviorally relevant information from complex environments and provide inspiration for creating bio-mimetic computer/robotic systems that can learn rapidly with low energy requirements”.

The insect mushroom body is also a versatile neural circuit crucial for encoding and processing odor information. Odor signals are relayed through the antenna, antennal lobe, and mushroom body calyx before reaching the mushroom body lobes. To better understand how Kenyon cells – the intrinsic mushroom body neurons - encode both odor identity and intensity, Lazar et al. developed a computational model called the “Odorant Encoding Machine,” simulating the olfactory stages based on *Drosophila*’s neural architecture. Their model revealed mechanisms that reduce response variability linked to odor concentration, effectively separating odor identity from intensity. They proposed a “first spike sequence code” used by Kenyon cells for efficient memory storage. This research deepens our understanding of sensory processing and highlights how studying insect brains can uncover general principles of neural computation.

Jürgensen et al. applied a computational modeling approach to explore how neural circuits might solve complex higher-order learning tasks. They also focused on the well-characterized mushroom body circuit in the *Drosophila* brain. Their findings revealed multiple ways this circuit could support second-order associative learning, each with distinct advantages and limitations. This study highlights the power of theoretical models in shaping experimental research and generating testable hypotheses in neuroscience.

In summary, this Research Topic highlights diverse studies exploring various aspects of learning and memory in invertebrate brains. The Research Topic demonstrates how invertebrate models can address a wide range of scientific questions, from basic molecular and cellular mechanisms to learning psychology, the toxicological effects of insecticides, robotic navigation, and computational models of information processing. This versatility reflects the immense diversity of invertebrate species, their specialized brains, and complex adaptive behaviors. Invertebrates thus offer a fertile ground for advancing our understanding of learning and memory across biological and technological domains.

